# Estrogen Receptor 1 (ESR1) and the Wnt/β-Catenin Pathway Mediate the Effect of the Coumarin Derivative Umbelliferon on Bone Mineralization

**DOI:** 10.3390/nu14153209

**Published:** 2022-08-05

**Authors:** Letizia Pelusi, Domitilla Mandatori, Nadia Di Pietrantonio, Francesco Del Pizzo, Pamela Di Tomo, Natalia Di Pietro, Roberto Buda, Salvatore Genovese, Francesco Epifano, Assunta Pandolfi, Serena Fiorito, Caterina Pipino

**Affiliations:** 1 Department of Medical, Oral and Biotechnological Sciences, Center for Advanced Studies and Technology-CAST, University G. D’Annunzio of Chieti-Pescara, 66100 Chieti, Italy; 2 Department of Neurosciences, Imaging and Clinical Sciences, Center for Advanced Studies and Technology-CAST, University G. D’Annunzio of Chieti-Pescara, 66100 Chieti, Italy; 3 Department of Medicine and Aging Sciences, University “G. D’Annunzio” Chieti-Pescara, 66100 Chieti, Italy; 4 Department of Pharmacy, University “G. D’Annunzio” of Chieti-Pescara, 66100 Chieti, Italy

**Keywords:** osteoblast, osteoclast, osteoporosis, coumarin derivatives, umbelliferon, bone mineralization, 3D culture

## Abstract

Bone physiology is regulated by osteoblast and osteoclast activities, both involved in the bone remodeling process, through deposition and resorption mechanisms, respectively. The imbalance between these two phenomena contributes to the onset of bone diseases. Among these, osteoporosis is the most common metabolic bone disorder. The therapies currently used for its treatment include antiresorptive and anabolic agents associated with side effects. Therefore, alternative therapeutic approaches, including natural molecules such as coumarin and their derivatives, have recently shown positive results. Thus, our proposal was to investigate the effect of the coumarin derivative umbelliferon (UF) using an interesting model of human osteoblasts (hOBs) isolated from osteoporotic patients. UF significantly improved the activity of osteoporotic-patient-derived hOBs via estrogen receptor 1 (ESR1) and the downstream activation of β-catenin pathway. Additionally, hOBs were co-cultured in microgravity with human osteoclasts (hOCs) using a 3D system bioreactor, able to reproduce the bone remodeling unit in bone loss conditions in vitro. Notably, UF exerted its anabolic role by reducing the multinucleated cells. Overall, our study confirms the potential efficacy of UF in bone health, and identified, for the first time, a prospective alternative natural compound useful to prevent/treat bone loss diseases such as osteoporosis.

## 1. Introduction

Bone is a dynamic tissue undergoing constant remodeling and turnover through a complex process in which the old or damaged bone is replaced by new bone. This phenomenon, known as the bone remodeling process, regulates the mineral contents in tissues responsible for maintaining bone strength and resistance [1,2]. The main cells involved in this process are the osteoblasts, specialized in the synthesis of bone matrix, and the osteoclasts, responsible for bone resorption [3]. The imbalance in the activity of these two types of cells leads to the development of several skeletal diseases, such as osteoporosis and osteopetrosis [4]. The latter is a rare genetic bone disease characterized by a reduction in osteoclast activity, resulting in an increase in bone mass with a paradoxical decrease in bone strength [5]. In contrast, osteoporosis is a systematic skeletal disease characterized by low bone mineral density and damage to the bone microstructure which, in turn, leads to a susceptibility to skeletal fractures [6]. This is caused by a higher activity of bone resorption by osteoclasts or by an impairment of osteoblast bone matrix deposition. The pathogenesis of osteoporosis is multifactorial and takes into account genetics and systemic factors. Furthermore, it is well known that estrogens play a key role in the regulation of bone mass, and their deficiency is one of the major causes of postmenopausal osteoporosis [7,8].

To date, osteoporosis is treated using antiresorptive or anabolic agents [9,10]. The former act by slowing down bone resorption, and bisphosphonates represent the main class used [11]. In addition, new antiresorptive drugs have been developed, including raloxifene (a selective estrogen receptor modulator), strontium ranelate (a calcium-sensing receptor activator), and denosumab (a human monoclonal antibody to the receptor activator of nuclear factor-κB ligand (RANKL), which blocks its binding to RANK), which are generally used for postmenopausal osteoporosis treatment. On the other hand, the full-length parathyroid hormone (PTH 1-84) or its N-terminal fragment and teriparatide (PTH 1-34) are used for their anabolic capacity [12].

Although all these treatments have shown encouraging results in restoring bone health, they are associated with several side effects, such as osteonecrosis of the jaw, atypical fractures of the femur, cardiovascular events, thromboembolism, esophageal stimulation, breast and uterine cancer, muscle cramps and worsening of menopause symptoms [13].

Therefore, in recent years, researchers have shifted their focus to the use of natural derivatives and their role in bone healing [14,15], since the use of natural compounds may be more suitable for long-term treatment, with fewer side effects compared to the pharmacological approaches currently adopted [16,17]. In this regard, we recently evaluated the role of menaquinone-4 in a three-dimensional co-culture system of human osteoblasts (hOBs) and osteoclast precursors from osteoporotic patients, demonstrating its capability in improving bone mineralization [18]. Notably, great attention has recently been paid to coumarin compounds and their derivatives. These are a class of secondary metabolites widely present in plants, citrus fruits, and legumes, characterized by a low molecular weight, ease of synthesis, and high bioavailability [19]. In addition, thanks to their structure, coumarins show a variety of pharmacological properties, such as antibacterial, antifungal, anti-inflammatory, antioxidant, anticoagulant, and antitumor effects [20]. Interestingly, several studies have investigated a possible role of coumarins in bone maintenance, highlighting an inhibitory effect on osteoclast activity [21,22,23,24]. Furthermore, a role in promoting osteogenic stem cell differentiation was also demonstrated [25], albeit no studies to date have evaluated the effects of coumarin compounds on primary human osteoblasts. Recently, we demonstrated that naturally occurring coumarins, such as aurapten, interact with estrogen receptor 1 (ESR1) [26], which is the main estrogen subtype resident in bone tissue, playing an essential role in hOB activity [27].

For this reason, the aim of this study was to assess the potential ESR1-mediated effects of coumarin derivatives on the bone mineralization process by using an interesting model of human primary osteoblasts isolated from osteoporotic patients. The occurring compounds umbelliferon (UF), 7-isopentenyloxycoumarin (7-ISO), auraptene (AU), and umbelliprenin (UP) were tested. Finally, through a 3D co-culture model composed of hOBs and human osteoclasts (hOCs), the effect of UF on bone remodeling activity was also investigated.

## 2. Materials and Methods

### 2.1. Experimental Plan

**Figure 1:**
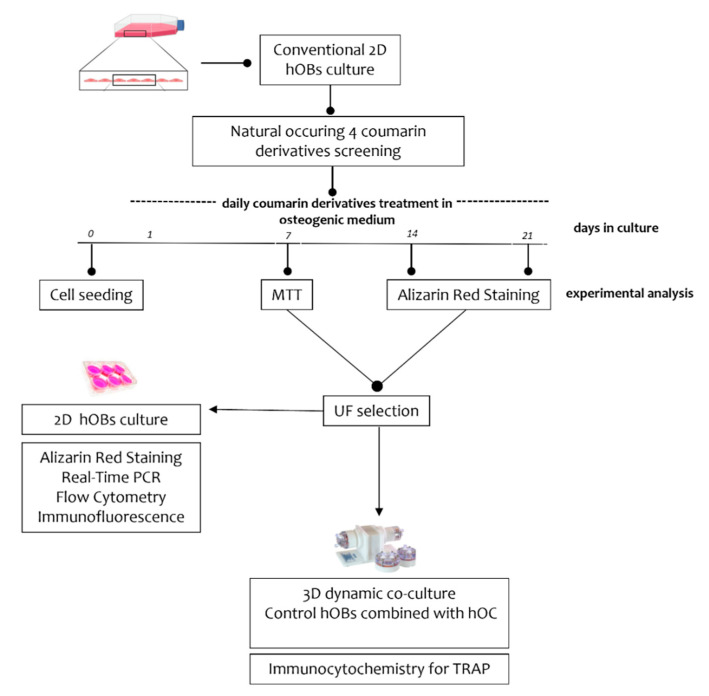
Experimental plan: hOBs were treated or not with 4 different coumarin derivatives—umbelliferon (UF), 7-Isopentenyloxycoumarin (7-ISO), aurapten (AU), or umbelliprenin (UP)—for 7, 14 or 21 days. After a screening by performing MTT assay and Alizarin Red staining, only UF was selected for further analysis. The effects of UF on bone mineralization were evaluated by performing Alizarin Red staining, real-time PCR, flow cytometry, and immunofluorescence analyses. In addition, TRAP staining was performed in a 3D co-culture system composed of control hOBs and hOCs in order to reproduce the bone remodeling unit under bone loss conditions in vitro.

As reported in Figure 1, employing a 2D culture system, hOBs isolated from osteoporotic patients (*n* = 10, median age 80.3 ± 11.9) were used to test the effects of the four different coumarin derivatives umbelliferon (UF), aurapten (AU), 7-isopentenyloxycoumarin (7-ISO) and umbelliprenin (UP) (Supplementary Figure S1), whose natural sources and chemical synthetic methods have been recently reviewed. In detail, UF was purchased commercially (Sigma-Aldrich, St. Louis, MO, USA), while 7-ISO, AU, and UP were obtained via chemical synthesis following the route reported in [28,29,30,31].

For some experiments, tamoxifen (5 µM in DMSO, Sigma-Aldrich, St. Louis, MO, USA) was added every other day for the duration of the treatment.

Based on the experimental plan, hOBs were induced to bone mineral matrix deposition by using a standard osteogenic medium (OM) [32]. During the mineralization induction, osteoporotic-patient-derived hOBs were treated with the molecules at different concentrations (0.1-1-10-50 µM) dissolved in dimethyl sulfoxide (DMSO) and added every day for up to 21 days. After a first screening of the four coumarins based on cell metabolic activity and bone matrix deposition, UF was selected for further analysis in 2D culture. Finally, employing an innovative 3D co-culture model able to reproduce a bone loss condition composed of hOBs and hOCs in vitro, the effect of UF on osteoclasts was also evaluated. For all experiments, cells were used between passages 3 and 5.

### 2.2. Cell Cultures

To perform 2D culture experiments, cryopreserved hOBs—isolated through spontaneous migration from bone fragments discarded during femoral surgery of patients undergoing osteoporosis-related femoral neck fracture—were used. Several clinical and biochemical parameters in these patients were previously evaluated to assess their osteoporotic condition [18]. All procedures were in accordance with the principles of the Declaration of Helsinki and the ethical standards of the Institutional Committee on Human Experimentation (Reference Number: N°24_22.10.2020). The protocol was approved by the Institutional Review Board of “G. d’Annunzio University” of Chieti-Pescara, and informed consent was signed by each participating subject.

Due to the difficulties of obtaining enough bone tissue from osteoporotic subjects to carry out 3D culture experiments, hOBs and hOCs from healthy subjects were used. 

As for the hOBs obtained from osteoporotic patients, control hOBs were isolated through spontaneous migration from bone fragments obtained—with signed informed consent—from healthy subjects undergoing traumatic orthopedic surgery (*n* = 3; median age 40 years). 

In parallel, to obtain hOCs, peripheral blood mononuclear cells (PBMCs) were isolated from peripheral blood of healthy volunteers recruited after giving informed consent (*n* = 3; median age 40 ± 6.11). Cells were separated by Histopaque^®®^-1077 (Sigma-Aldrich, St. Louis, MO, USA) using a standardized protocol. Human monocyte cells (hMCs) were purified from 2.5 × 10^6^ cells/cm^2^ by adhesion selection on polystyrene plates. Then, after 3 h of incubation (5% CO_2_, 37 °C), the flasks were rinsed to remove non-adherent cells. Differentiation of osteoclasts from isolated hMCs was induced by adding 50 ng/mL M-CSF and 30 ng/mL RANKL (PeproTech EC Ltd., London, UK) in culture medium.

In order to develop a 3D model able to mimic bone loss conditions in vitro, a microgravity condition was applied [33,34] in the RCCS-4TM bioreactor (Synthecon™, Inc., Houston, TX, USA). In detail, as previously described [18], control hOBs and hOCs were mixed (2:1 ratio, 2 × 10^6^ and 1 × 10^6^) in 2 mL of High-Aspect Ratio Vessels (HARV™; Synthecon™, Inc., Houston, TX, USA) filled with control medium (CTRL) composed of low-glucose Dulbecco’s modified Eagle Medium (DMEM L-GLU, Gibco-Life Technologies, Waltham, MA, USA), 10% fetal bovine serum (FBS, Gibco-Life Technologies, Waltham, MA, USA), 1% l-glutamine (L-GLU), and 1% penicillin/streptomycin (P/S) (Sigma-Aldrich, St. Louis, MO, USA). The RCCS-4TM rotary bioreactor was set up at 4 rpm (ground-based gravity) in the incubator (5% CO_2_, 37 °C) to promote 3D culture formation. After 2 days, the bioreactor was set up to 16 rpm (microgravity), and the CTRL medium was replaced with osteogenic medium (OM). 

### 2.3. 3-(4, 5-Dimethylthiazolyl-2-yl)-2, 5-Diphenyltetrazolium Bromide (MTT) Cell Metabolic Activity

The effects of coumarin derivatives (AU, UF, 7-ISO, and UP) on hOBs’ metabolic activity was assessed by the MTT assay (cat. M211281G, Sigma-Aldrich, St. Louis, MO, USA). According to the manufacturer’s instructions, cells were seeded in a 96-well plate at a density of 5000 cells/well (20,000 cells/mL). Following 7 days of treatment, the spectrophotometric analysis was carried out at a wavelength of 540 nm using a microplate absorbance reader (SpectraMax 190, Molecular Devices, San Jose, CA, USA).

### 2.4. Alizarin Red Staining

Alizarin Red staining (ARS; Sigma-Aldrich, St. Louis, MO, USA) experiments were performed to quantify the level of bone matrix deposition; hOBs were seeded in 6-well plates at a density of 80,000 cells/well and treated for 14 and 21 days. After fixing with 10% formaldehyde, hOBs were stained with ARS solution (40 mM, pH 4.2) for 20 min at room temperature (RT). The quantification was performed at a wavelength of 405 nm using a microplate reader (SpectraMax 190; Molecular Devices, San Jose, CA, USA).

### 2.5. Real-Time PCR (RT-PCR)

Total RNA was extracted using TRIzol reagent (Thermo Fisher Scientific, Waltham, MA, USA) following the manufacturer’s instructions. The RNA concentration and quality were measured using the NanoDrop 2000c Spectrophotometer (Thermo Fisher Scientific, Waltham, MA, USA). cDNA was synthetized by using the High-Capacity cDNA Reverse Transcription Kit (Thermo Fisher Scientific, Waltham, MA, USA). The TaqMan Universal PCR Master Mix (Thermo Fisher Scientific, Waltham, MA, USA) and TaqMan Gene Expression Assay (Thermo Fisher Scientific, Waltham, MA, USA) probes for human alkaline phosphatase (ALP; Hs01029144_m1), human osteopontin (OPN, Hs00959010_m1), human collagen type 1 (COL1a1, Hs00164004_m1), human osteocalcin (OC, Hs01587813_g1), human estrogen receptor 1 (ESR1, Hs01046816_m1), human catenin beta 1 (CTNBB1, Hs00355045_m1), and human glyceraldehyde-3-phosphate dehydrogenase (GAPDH, Hs02786624_g1) were used according to the manufacturer’s instructions. The relative gene expression was calculated by using the comparative 2-ΔΔCT method.

### 2.6. Flow Cytometry 

Protein expression of ALP, OPN, OC, and COL1a1 was evaluated by flow cytometry analysis, following a previous protocol [35]. Samples were processed using a FACS Canto II flow cytometer (BD Biosciences, San Jose, CA, USA), and data were analyzed using FACSDiva v6.1.3, IDEAS software (BD Biosciences, San Jose, CA, USA), and FlowJo v8.3.3 software (Tree Star Inc., Ashland, OR, USA). Data are indicated as a mean fluorescence intensity (MFI) ratio calculated by dividing the MFI of positive events by the MFI of negative events (MFI of secondary antibody).

### 2.7. Immunofluorescence

β-Catenin expression was evaluated by immunofluorescence experiments. In detail, hOBs were seeded on sterile glass coverslips in 24-well plates and, following 24 h and 72 h of coumarin treatment, the cells were fixed in 4% paraformaldehyde (10 min, RT), permeabilized with Triton (0.1%; 10 min RT), and stained with β-catenin (cat. ab6302, Abcam), primary antibody (1:200), and Alexa Fluor 488 anti-rabbit secondary antibody (cat. A11034, Invitrogen, Thermo Fisher Scientific, Waltham, MA, USA). DAPI staining was used to determine the nuclei. Cells were observed through a confocal microscope (Zeiss LSM-800, Carl Zeiss Meditec AG, Oberkochen, Germany). Data, calculated as β-catenin fluorescence intensity, were obtained by analyzing at least three different fields for each image with ImageJ software (NIH, US, ImageJ software).

### 2.8. Immunocytochemistry

Cell aggregates were attached to SuperFrost^®®^ Plus Microscope Slides (Thermo Fisher Scientific, Waltham, MA, USA) using a Cytospin centrifuge. Next, they were blocked with Protein Block (Agilent Technologies, Santa Clara, CA, USA) for 10 min at RT, incubated with primary anti-TRAP (tartrate-resistant acid phosphatase) antibody (PA5-116970, Invitrogen), and diluted 1:100 in antibody diluent (Agilent Technologies, Santa Clara, CA, USA) for 1 h, and then with the anti-rabbit secondary antibody (111-065-003, Jackson ImmunoResearch, Cambridge House, St. Thomas Place, UK) for 30 min. Immunoreactive antigens were detected using streptavidin peroxidase (Thermo Fisher Scientific, Waltham, MA, USA) and the DAB Chromogen System (Agilent Technologies, Santa Clara, CA, USA). After chromogen incubation, the slides were counterstained in hematoxylin (Bio-Optica, Milano, IT), and images were acquired using a Leica DMRD optical microscope (Leica, Wetzlar, DE, Germany).

### 2.9. Statistical Analysis

Data were expressed as the mean ± standard error (SEM). Statistical significance was analyzed using GraphPad Prism Software Analysis (version 9, San Diego, CA, USA) via one-way ANOVA followed by Tukey’s post hoc test. The α value was set at 0.05.

## 3. Results

### 3.1. Effects of Coumarin Derivatives on hOBs’ Viability and Bone Matrix Deposition Ability

As reported in our previous study [18], the cells were obtained from osteoporotic patients and characterized for their morphology (immunofluorescence; Supplementary Figure S2A), their capacity to release mineralized bone matrix (Alizarin Red staining; Supplementary Figure S2B), and the expression of typical bone markers such as ALP, Runx2, OC, OP, and COL1a1 (flow cytometry; Supplementary Figure S2C). 

**Figure 2:**
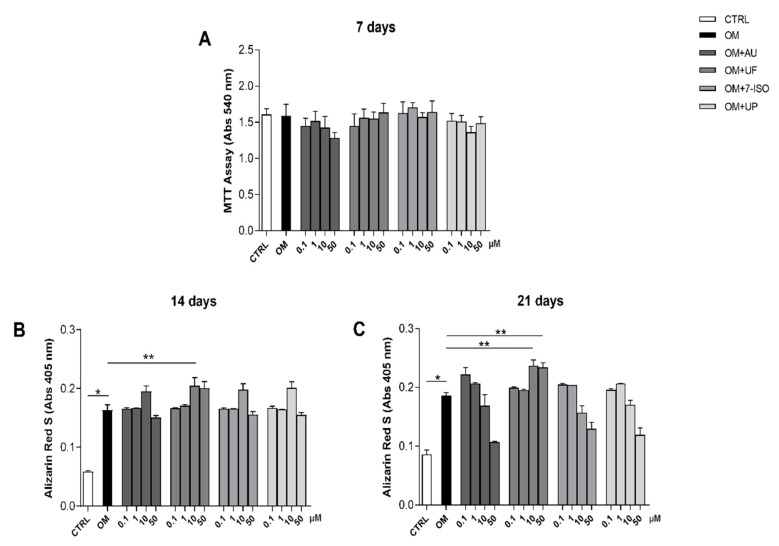
Cells’ metabolic activity and bone matrix deposition: (**A**) Effects of coumarin derivatives (AU, UF, 7-ISO, and UP; 0.1-1-10-50 µM) on hOBs’ metabolic activity following 7 days of culture. Mineral matrix deposition quantification after (**B**) 14 and (**C**) 21 days of treatment with the molecules. Results are shown as the mean ± error standard (SEM) (*n* ≥ 3); * *p* < 0.05 vs. CTRL; ** *p* < 0.05 vs. OM.

The effects of the four coumarin derivatives (UF, AU, 7-ISO, and UP) on hOBs’ metabolic activity were investigated at different concentrations (0.1-1-10-50 µM). Interestingly, after 7 days of treatment, none of the tested molecules significantly affected the metabolic activity of the hOBs (Figure 2A). It should be noted that the activity of osteoblasts in terms of bone mineral matrix deposition was differently modulated by the four tested coumarins, as reported in Figure 2B,C.

Interestingly, among the coumarin derivatives, only the UF (10 µM) induced a significant increase in mineral matrix deposition compared to the OM condition (*p* < 0.05), at both 14 and 21 days. Based on these results, we selected UF at 10 µM for the subsequent experiments.

### 3.2. hOBs’ Responsiveness to UF and Estrogen Receptor 1 Involvement

**Figure 3:**
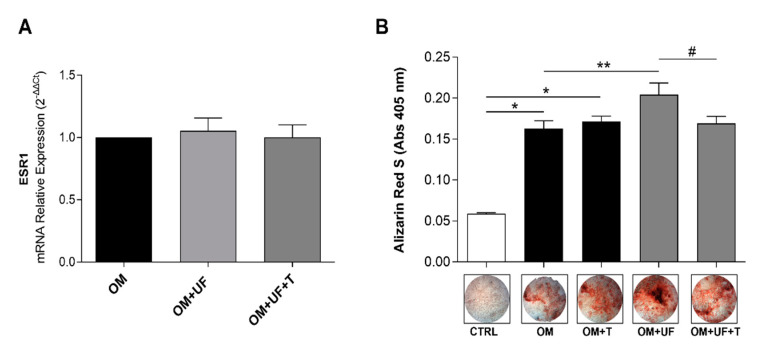
mRNA expression of ESR1, and effects of UF treatment on 2D-cultured hOBs: (**A**) Effect of UF on ESR1 expression following 14 days of treatment. Data are shown as the mean ± standard (error SEM). (**B**) hOBs were cultured for 14 days with CTRL medium and OM in the presence or absence of UF (10 µM) and tamoxifen (5 µM). Quantitative analyses of ARS were performed, and representative images were reported. Results are expressed as the mean ± standard error (SEM) (*n* ≥ 3); * *p* < 0.05 vs. CTRL; ** *p* < 0.05 vs. OM; # *p* < 0.05 vs. OM + UF + T.

In order to investigate whether the effect of UF in our model of primary hOBs was mediated by ESR1, we firstly confirmed the expression of ESR1 in our cellular model by real-time PCR (Figure 3A), finding that its expression was not influenced by UF treatment in the presence or absence of 5 µM tamoxifen—a selective estrogen receptor modulator (SERM). Then, we interestingly found that the augmented mineral matrix deposition induced by UF treatment was significantly reverted by the presence of tamoxifen, thus suggesting a possible role of ESR1 (Figure 3B; *p* < 0.05 vs. OM + UF).

**Figure 4:**
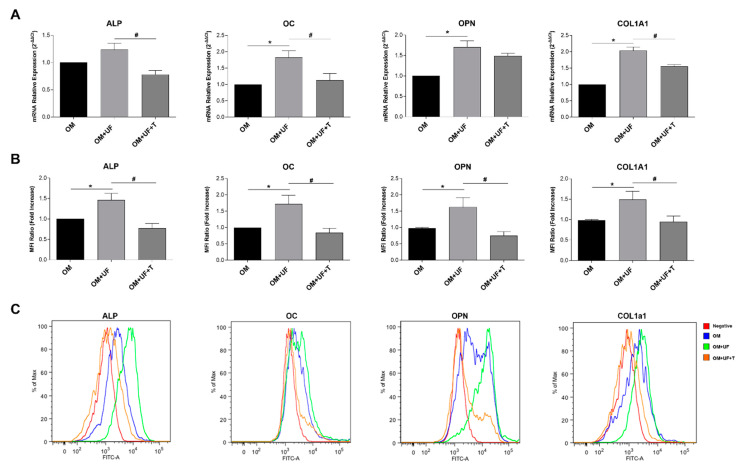
Effects of UF treatment on mRNA and protein expression of typical bone markers: Effects of UF treatment on the (**A**) mRNA expression and (**B**) protein expression of the specific osteoblast markers ALP, OC, OPN, and COL1a1, analyzed by RT-qPCR and flow cytometry, respectively. (**C**) Representative flow cytometry histograms of the specific osteoblast markers ALP, OC, OPN, and COL1a1. Results are shown as the mean ± standard error (SEM) (*n* ≥ 3); * *p* < 0.05 vs. OM # *p* < 0.05 vs. OM + UF + T (10 µM).

Accordingly, as clearly shown in Figure 4, the increased mRNA levels (Figure 4A) and protein expression (Figure 4B,C) of ALP, OC, OPN, and COL1a1 induced by UF (10 µM; *p* < 0.05 vs. OM) were significantly inhibited by tamoxifen treatment (5 µM; *p* < 0.05 vs. OM).

These data significantly reinforce the upstream role of ESR1 in the mechanism leading to UF-enhanced mineral matrix deposition in cultured hOBs.

### 3.3. β-Catenin Pathway

**Figure 5:**
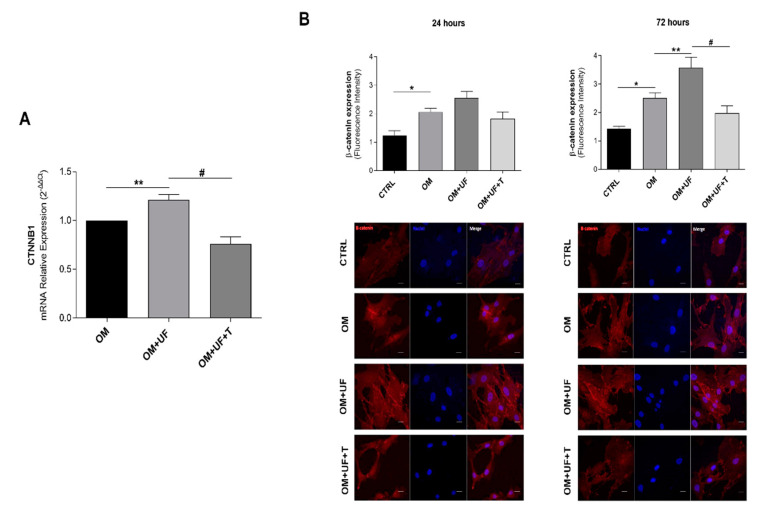
Effect of UF treatment on β-catenin expression: Effects of UF treatment on the (**A**) mRNA expression and (**B**) protein expression of β-catenin, analyzed by RT-PCR (CTNBB1) and immunofluorescence, respectively. Results are shown as the mean ± standard error (SEM) (*n* ≥ 3); * *p* < 0.05 vs. CTRL; ** *p* < 0.05 vs. OM; # *p* < 0.05 vs. OM + UF + T (10 µM).

Since it is well recognized that ESR1 is implicated in the modulation of the Wnt/β-catenin pathway in hOBs [8,36], we evaluated the expression of β-catenin (*CTNNB1* gene) by RT-PCR. Remarkably, we found that UF significantly increased the expression of β-catenin, and this effect was significantly reverted by 5 µM tamoxifen (Figure 5A). In addition, UF-treated cells showed a significant increase in β-catenin protein levels (semi-quantified by immunofluorescence analysis, *p* < 0.05 vs. OM), which were significantly reduced following 24 and 72 h tamoxifen treatment (*p* < 0.05 vs. OM + UF), suggesting that the role exerted by UF in cultured hOBs can be mediated by the ESR1/β-catenin pathway (Figure 5B).

### 3.4. UF’s Effects on the hOBs/hOCs 3D Co-Culture System

**Figure 6:**
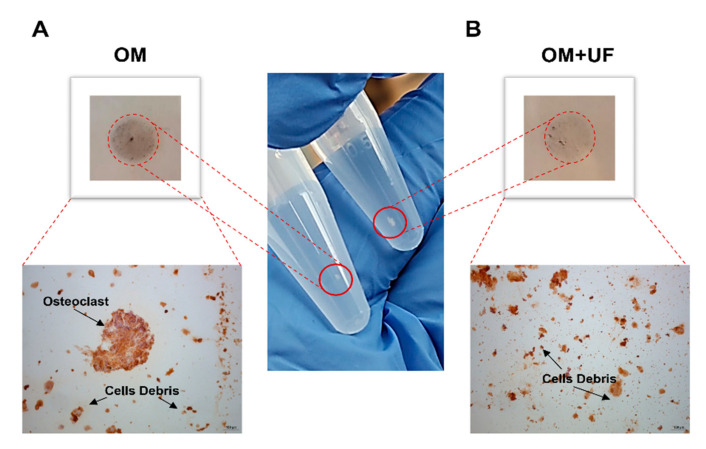
Effects of UF on the control hOBs/hOCs 3D-DyC Co-Culture System: hOCs and hOBs were co-cultured in microgravity in a 3D system bioreactor (RCCS-4TM) for 14 days in the (**A**) presence or (**B**) absence of UF (10 µM). Representative images of cell aggregates and TRAP analyses were reported.

Finally, co-culturing control hOBs/hOCs in a microgravity 3D-DyC System, we developed a 3D system mimicking the bone loss condition in vitro. Therefore, cells were co-cultured in OM 3D dynamic conditions in the presence or absence of UF (10 µM). As shown in Figure 6, following 14 days of co-culture, the UF-treated aggregates showed a size and density more appreciable than those of the untreated aggregates.

Subsequently, the level of TRAP as specific osteoclast marker was analyzed by immunohistochemical analysis, demonstrating decreased TRAP staining in UF-treated aggregates compared to untreated ones. In accordance with this, the untreated aggregates showed appreciable multinucleated cells (Figure 6, Panels A and B) compared to UF-treated ones, suggesting that UF is able not only to modulate osteoblast activity, but also to reduce the osteoclasts’ differentiation and activity. 

## 4. Discussion

In the present study, we investigated the role of the coumarin compound UF on an explanatory in vitro model of human primary osteoblasts obtained from osteoporotic patients. Initially, we tested the biological activity of the coumarin compounds UF, AU, 7-ISO, and UP, evaluating the expression of osteogenic markers and formation of bone mineral matrix. Interestingly, only UF (10 µM) induced a significant increase in mineral matrix deposition. Therefore, we selected this molecule for further detailed investigation, and predicted its possible interaction with ESR1—the main estrogen subtype expressed in hOBs, playing a crucial role in their anabolic activity [37]. Additionally, by using tamoxifen (a selective modulator of ESR1), we investigated the potential involvement of ESR1 in the process of mineral matrix deposition and expression of osteogenic markers mediated by UF in these cells.

Today, coumarin and its derivatives, although mostly studied for their anti-inflammatory and antimicrobial properties, have shown positive effects on bone metabolism. Some compounds—such as 5-hydroxy aurapten (5-HA), heraclenin, aesculetin, imperatorin, bergapten, osthole, and isopsoralen—have been investigated for promoting the differentiation of stem cells into osteoblasts, and their possible implication in the bone remodeling process [23,24,25,38,39,40,41]. Interestingly, the coumarin 5′-HA was able to stimulate the differentiation of bone-marrow-derived mesenchymal stem cells into osteogenic cell lineages in a dose-dependent manner [25]. Moreover, the coumarin aesculetin was found to be beneficial in enhancing osteoblastic differentiation and matrix-vesicle-mediated collagen mineralization in MC3T3-E1 osteoblasts, thus indicating its usefulness in preventing bone pathologies or enhancing bone regeneration [39]. Certainly, this relevant literature allowed the identification of novel coumarin derivatives as potential osteoanabolic compounds. However, the role of these molecules has never been investigated in mature human osteoblasts. In this regard, to the best of our knowledge, this study provides the first evidence for the role of the coumarin UF in an in vitro model of hOBs obtained from osteoporotic patients.

To better understand the mechanism potentially involved in the osteogenic effects of UF and taking into account our previous findings showing the interaction of the coumarin aurapten with ESR1 [26], we investigated whether the effects of UF were mediated by ESR1 in a model of primary hOBs from osteoporotic patients. First, we confirmed the expression of ESR1 in our cellular model. Strikingly, the increased mineral matrix deposition induced by UF treatment, together with the improvement of the osteogenic markers analyzed, was significantly reduced when tamoxifen—a selective inhibitor of ESR1—was added, suggesting the involvement of ESR1 in this process. Since a growing body of literature has reported that the Wnt/β-catenin pathway is implicated in the mechanism by which osteoblasts and osteoclasts regulate bone architecture to maintain bone strength [42], to further investigate the mechanisms of UF in inducing mineralization of hOBs, we evaluated the β-catenin expression. Interestingly, we found that UF increased the expression of β-catenin, which was significantly reverted following treatment with 5 µM tamoxifen, reinforcing the finding that the effectiveness of UF requires ESR1. These data clearly suggest the involvement of the ESR1/β-catenin pathway in the UF-mediated hOBs’ mineralization. In detail, we found that the β-catenin expression was increased in all differentiated hOBs—mainly at the membrane level—following UF treatment. This was probably due to the role of β-catenin as an integral structural component of cadherin-based adherent junctions [43], which increase following the bone mineral matrix deposition. 

To closely mimic the bone remodeling unit, we developed an innovative 3D experimental model combining control hOBs and hOCs. However, a limitation should be considered when interpreting these findings. Indeed, for this experiment, due to the large number of cells required (2 million cells for each condition) and the difficulty in the recruitment of osteoporotic patients, hOBs of healthy individuals were used and subjected to the abovementioned protocol to mimic the bone loss condition in vitro. Nevertheless, this study is novel in several respects. Firstly, for this study, we used the relevant model of human osteoblasts from osteoporotic patients, which have unquestionable attractiveness in terms of cell behavior closer to the in vivo patient niche. Secondly, we further confirmed our previous observation regarding the interaction of coumarin compounds with ESR1 and investigated the UF compound in depth. Thirdly, we elucidated a novel role of UF as a protector against osteoporosis by activating the ESR1/β-catenin pathway. The data obtained indicate that UF was able not only to modulate osteoblast activity, but also to reduce the activity of osteoclasts. These results are supported by other studies on coumarin derivatives, such as one in which the effect of UF on the reduction of lipopolysaccharide (LPS)-induced inflammatory bone loss in vivo was investigated [22]. As a result, UF may be considered as a potential therapeutic agent for bone loss diseases associated with abnormal osteoblast and osteoclast function. 

## 5. Conclusions

Overall, these findings add new clues for the treatment of patients with osteoporosis and may have broader implications for estrogen-associated diseases. Our research provides scientific support for a future safely moderated intake of UF-rich food or UF as a supplement in the daily diet to better prevent osteoporosis.

## Data Availability

The data analyzed in this study are not publicly available, because individual privacy may be compromised. Interested groups may contact Caterina Pipino (caterina.pipino@unich.it) to request permission to access these datasets.

## Supplementary Materials

The following supporting information can be downloaded at: https://www.mdpi.com/article/10.3390/nu14153209/s1, Figure S1. Chemical structure of natural occurring coumarin derivatives such as Umbelliferon (UF), 7-Isopentenyloxycoumarin (7-ISO), Aurapten (AU), Umbelliprenin (UP). Figure S2: Characterization of hOBs.

## References

[1] Pasqualetti S., Banfi G. (2012). The Zebrafish Scale as Model to Study the Bone Mineralization Process. J. Mol. Histol..

[2] Bala Y., Farlay D. (2013). Bone Mineralization: From Tissue to Crystal in Normal and Pathological Contexts. Osteoporos. Int..

[3] Dirckx N., Moorer M.C. (2019). The Role of Osteoblasts in Energy Homeostasis. Nat. Rev. Endocrinol..

[4] Baeuerlein E., Behrens P. (2007). Handbook of Biomineralization..

[5] Teti A., Econs M.J. (2017). Osteopetroses, Emphasizing Potential Approaches to Treatment. Bone.

[6] Appelman-Dijkstra N.M., Papapoulos S.E. (2015). Modulating Bone Resorption and Bone Formation in Opposite Directions in the Treatment of Postmenopausal Osteoporosis. Drugs.

[7] Streicher C., Heyny A. (2017). Estrogen Regulates Bone Turnover by Targeting RANKL Expression in Bone Lining Cells. Sci. Rep..

[8] Xia H., Liu J. (2021). Integrated Strategy of Network Pharmacological Prediction and Experimental Validation Elucidate Possible Mechanism of Bu-Yang Herbs in Treating Postmenopausal Osteoporosis via ESR1. Front. Pharmacol..

[9] Bilezikian J.P. (2007). Anabolic Therapy for Osteoporosis. Women’s Health.

[10] Seeman E., Martin T.J. (2019). Antiresorptive and Anabolic Agents in the Prevention and Reversal of Bone Fragility. Nat. Rev. Rheumatol..

[11] Chen J.S., Sambrook P.N. (2012). Antiresorptive Therapies for Osteoporosis: A Clinical Overview. Nat. Rev. Endocrinol..

[12] Adami S. (2008). Full Length Parathyroid Hormone, PTH(1-84), for the Treatment of Severe Osteoporosis in Postmenopausal Women. Curr. Med. Res. Opin..

[13] Skjødt M.K., Frost M. (2019). Side Effects of Drugs for Osteoporosis and Metastatic Bone Disease. Br. J. Clin. Pharmacol..

[14] Miranda L.L., Guimarães-Lopes V.D.P. (2019). Plant Extracts in the Bone Repair Process: A Systematic Review. Mediat. Inflamm..

[15] Putnam S.E., Scutt A.M. (2007). Natural Products as Alternative Treatments for Metabolic Bone Disorders and for Maintenance of Bone Health. Phytother. Res.

[16] Park E., Kim J. (2020). Scopolin Attenuates Osteoporotic Bone Loss in Ovariectomized Mice. Nutrients.

[17] Zhang T., Zhong S. (2018). Estrogenic Properties of Coumarins and Meroterpene from the Fruits of Cullen Corylifolium: Experimental and Computational Studies. Phytochemistry.

[18] Mandatori D., Penolazzi L. (2021). Three-Dimensional Co-Culture System of Human Osteoblasts and Osteoclast Precursors from Osteoporotic Patients as an Innovative Model to Study the Role of Nutrients: Focus on Vitamin K2. Nutrients.

[19] Zhu J.J., Jiang J.G. (2018). Pharmacological and Nutritional Effects of Natural Coumarins and Their Structure–Activity Relationships. Mol. Nutr. Food Res..

[20] Peng X.-M., Damu G.L. (2013). Current developments of coumarin compounds in medicinal chemistry. Curr. Pharm. Des..

[21] Tavares S.J.S., Lima V. (2021). Bone Anti-Resorptive Effects of Coumarins on RANKL Downstream Cellular Signaling: A Systematic Review of the Literature. Fitoterapia.

[22] Kwak S.C., Baek J.M. (2019). Umbelliferone Prevents Lipopolysaccharide-Induced Bone Loss and Suppresses Rankl-Induced Osteoclastogenesis by Attenuating Akt-c-Fos-Nfatc1 Signaling. Int. J. Biol. Sci..

[23] Ma Y., Wang L. (2019). Osthole Inhibits Osteoclasts Formation and Bone Resorption by Regulating NF-ΚB Signaling and NFATc1 Activations Stimulated by RANKL. J. Cell. Biochem..

[24] Na W., Lee E.J. (2020). Aesculetin Inhibits Osteoclastic Bone Resorption through Blocking Ruffled Border Formation and Lysosomal Trafficking. Int. J. Mol. Sci..

[25] Abdallah B.M., Ali E.M. (2019). 5′-Hydroxy Auraptene Stimulates Osteoblast Differentiation of Bone Marrow-Derived Mesenchymal Stem Cells via a BMP-Dependent Mechanism. J. Biomed. Sci..

[26] Fiorito S., Epifano F. (2018). Natural Oxyprenylated Coumarins Are Modulators of Melanogenesis. Eur. J. Med. Chem..

[27] Schiavi J., Fodera D.M. (2021). Estrogen Depletion Alters Osteogenic Differentiation and Matrix Production by Osteoblasts in Vitro. Exp. Cell Res..

[28] Preziuso F., Genovese S. (2020). 7-Isopentenyloxycoumarin: What Is New across the Last Decade. Molecules.

[29] Faraone I., Russo D. (2021). Screening of in Vitro and in Silico α-Amylase, α-Glucosidase, and Lipase Inhibitory Activity of Oxyprenylated Natural Compounds and Semisynthetic Derivatives. Phytochemistry.

[30] Fiorito S., Epifano F. (2019). Biomolecular Targets of Oxyprenylated Phenylpropanoids and Polyketides. Prog. Chem. Org. Nat. Prod..

[31] Sarker S.D., Nahar L. (2017). Progress in the Chemistry of Naturally Occurring Coumarins. Prog. Chem. Org. Nat. Prod..

[32] Binder B.Y.K., Sagun J.E. (2015). Reduced Serum and Hypoxic Culture Conditions Enhance the Osteogenic Potential of Human Mesenchymal Stem Cells. Stem. Cell Rev. Rep..

[33] Nabavi N., Khandani A. (2011). Effects of Microgravity on Osteoclast Bone Resorption and Osteoblast Cytoskeletal Organization and Adhesion. Bone.

[34] Nagaraja M.P., Risin D. (2013). The Current State of Bone Loss Research: Data from Spaceflight and Microgravity Simulators. J. Cell. Biochem..

[35] Di Tomo P., Pipino C. (2013). Calcium Sensing Receptor Expression in Ovine Amniotic Fluid Mesenchymal Stem Cells and the Potential Role of R-568 during Osteogenic Differentiation. PLoS ONE.

[36] Gao Y., Huang E. (2013). Crosstalk between Wnt/β-Catenin and Estrogen Receptor Signaling Synergistically Promotes Osteogenic Differentiation of Mesenchymal Progenitor Cells. PLoS ONE.

[37] Almeida M., Laurent M.R., Claessens F., O’brien C.A., Bouillon R., Vanderschueren D., Manolagas S.C. (2017). Estrogens and Androgens in Skeletal Physiology and Patho-Physiology. Physiol. Rev..

[38] Liang Y., Xie L. (2021). Bergapten: A Review of Its Pharmacology, Pharmacokinetics, and Toxicity. Phytother. Res..

[39] Na W., Kang M.K. (2021). Aesculetin Accelerates Osteoblast Differentiation and Matrix-Vesicle-Mediated Mineralization. Int. J. Mol. Sci..

[40] Shanmugam H., Dharun V.N. (2019). Osteogenic Stimulatory Effect of Heraclenin Purified from Bael in Mouse Mesenchymal Stem Cells in Vitro. Chem. -Biol. Interact..

[41] Zhang T., Han W. (2019). Psoralen Accelerates Bone Fracture Healing by Activating Both Osteoclasts and Osteoblasts. FASEB J..

[42] D’Alimonte I., Lannutti A. (2013). Wnt Signaling Behaves as a “Master Regulator” in the Osteogenic and Adipogenic Commitment of Human Amniotic Fluid Mesenchymal Stem Cells. Stem. Cell Rev. Rep..

[43] Stamos J.L., Weis W.I. (2012). The Beta-Catenin Destruction Complex. EMBO J..

