# Supplementary material for: Estrogen Receptor 1 (ESR1) and the Wnt/β-Catenin Pathway Mediate the Effect of the Coumarin Derivative Umbelliferon on Bone Mineralization
Source: Nutrients. 2022 Aug 5;14(15):3209. doi: 10.3390/nu14153209 (PMC9370350; doi:10.3390/nu14153209)
Supplement: Supplementary file 1 [file nutrients-14-03209-s001.zip › nutrients-1835805-supplementary.pdf]

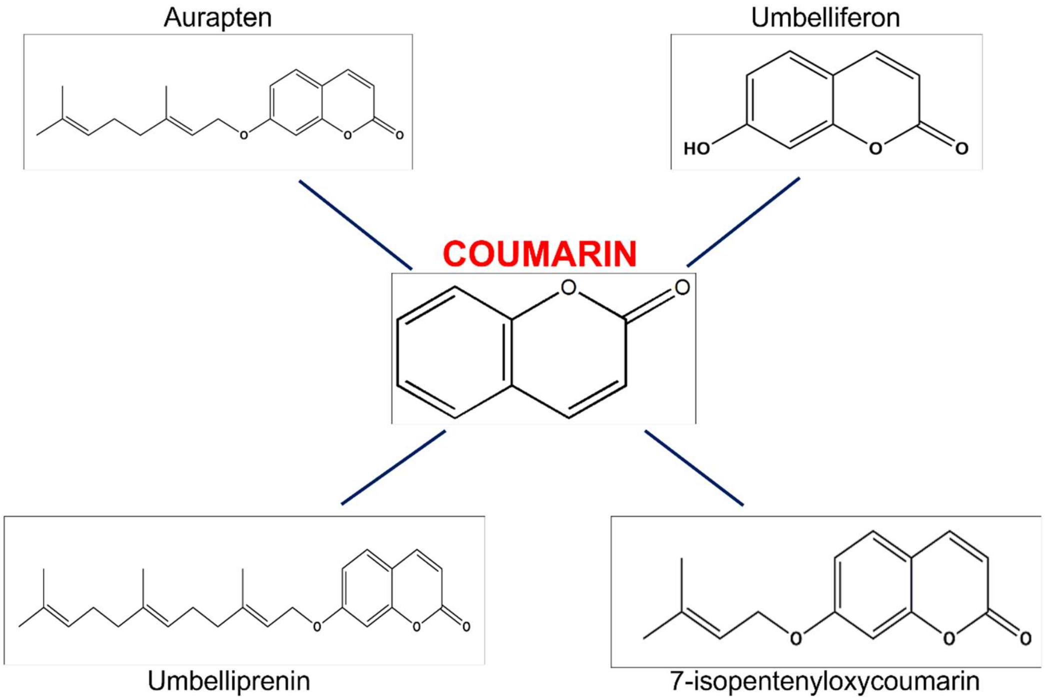

Figure S1. Chemical structure of natural occurring coumarin derivatives such as Umbelliferon (UF), 7-Isopentenyloxycoumarin (7-ISO), Aurapten (AU), Umbelliprenin (UP).

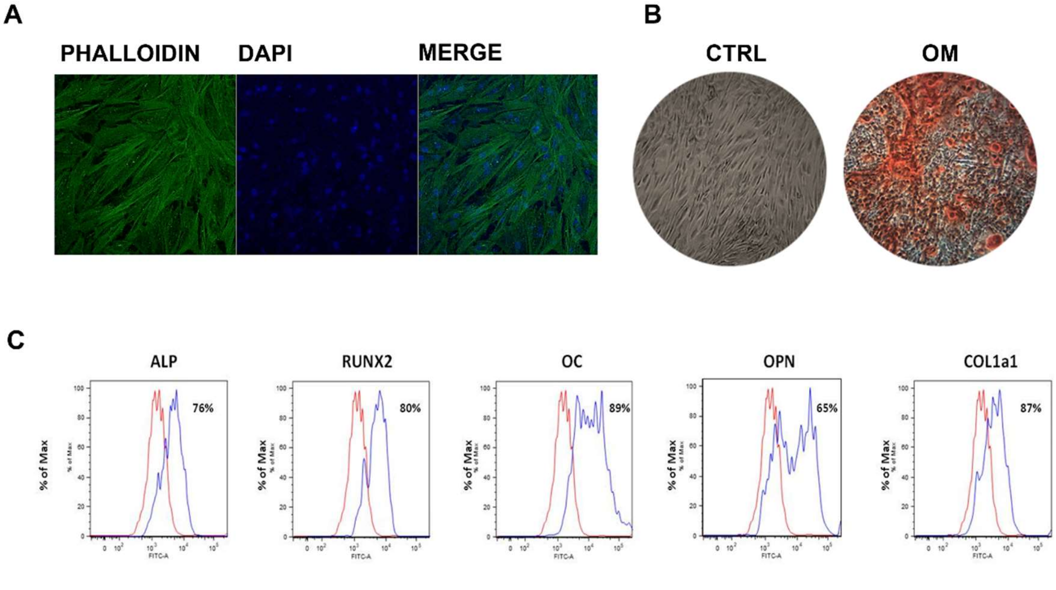

Figure S2. Characterization of hOBs. (**A**) Representative immunofluorescence images of isolated hOBs. (**B**) Bone matrix deposition evaluated by ARS staining (representative images). (**C**) Expression of typical osteoblastic markers (ALP,

Runx2, OC, OPN, and COL1a1) analyzed by flow cytometry (representative histograms). Red line: negative population. Blue line: positive population.
